# New York Surgical Society

**Published:** 1881-08

**Authors:** 


### NEW YORK SURGICAL SOCIETY.

Dr. H. Guleke presented a patient whose history was included in the communication he made to the society.

*Naso Pharyngeal Tumors.*—The following is an abstract of the three cases reported:

Case I.—William K——, aged seventeen years [presented to the society], has suffered for nearly a year from an impediment to breathing. The right side of his nose was nearly closed, and there was some catarrhal discharge. When moving, or when asleep, the boy was obliged to keep his mouth open. Unable to pronounce the nasal letters. Hearing unaffected. No headache. Examination by sunlight, through the aperture of the right nostril, revealed far back a bright red tumor with smooth surface.

The fauces were swollen, red, and covered with some mucus. Examination with the finger revealed that the pharynx was filled, on the right side especially, with a number of small and soft bodies, and a larger one that sprang from the upper wall and the outside of the pharynx. Free hemorrhage followed the examination, but it soon ceased. No clue to the origin of the disease could be obtained. The patient had occasionally had bleeding from that region, but not sufficient to render him anæmic, and he was well developed. Medical treatment had not afforded any benefit.

On the 17th of January, Dr. Guleke applied the galvano-caustic loop and removed several of the small growths and a portion of the large one. After the operation the patient could force more air through the nostril than previously. Dr. Sessel examined microscopically the portions removed and reported that they were adenoid vegetations. Dr. Guleke had gained a little more space by two more operations with the loop, but he finally regarded the instrument as inapplicable in that case, because it slipped from the cone-shaped vegetation before it had time to burn a groove in the tumor.

Case II.—A. G——, aged fifteen years, was sent to him by Dr. Simrock in July, 1875. The boy was well-developed, healthy-looking, but could not breathe through his nose. There was a slight discharge from the nose, and the patient complained of constant headache, especially on the left side, and had pain in the left orbit. His hearing was unimpaired. The fauces were swollen, and were thickly coated with greenish mucus. On examination with the finger a large, flat body was found just above the lower edge of the velum on the back wall of the pharynx, and a second tumor, softer and more cone shaped, closed the entrance to the left nostril. The history of the case shed no light upon the origin of the tumors. His speech was seriously affected.

On the 5th of August, 1875, Dr. Guleke, in the presence of Dr. Krackowizer and Dr. Simrock, removed with the galvano-caustic loop all of the first or lower tumor and the greater part of the second or upper one. He subsequently tried several times to remove the base of the upper tumor, but was unable to keep the loop in position properly. The tumor began to grow again, and he then scraped it off with a sharp spoon, and the disease had not returned.

Case III.—A. K——, aged eight years, came to the German Dispensary September 2, 1876. The boy was small for his age and pale, his eyes were dull and his mouth constantly open. His voice was without sound. His hearing was unaffected. Examination of the fauces revealed a tumor, over which the lower edge of the soft palate was stretched and pushed forward. The finger could enter only the naso pharyngeal space upon both sides of the tumor. The galvano-caustic loop was applied at once, and the tumor completely removed, and there had been no recurrence of the disease. The attachment of the tumor was in the middle of the posterior wall of the pharynx.

*Remarks.*—The description of these growths, first given by Dr. William Meyer, correspond with the conditions found in these cases; and even the absence of the most frequent symptom, the more or less aggravated conditions of the ear, Dr. Guleke thought, did not exclude them from Dr. Meyer's class of cases. Meyer had reported one hundred and seventy five cases, of which about forty-five had no affection of the ear. The most constant symptoms seemed to be the inflamed and swollen condition of the fauces and palate, especially the posterior arches. Then followed the affection of the speech and the consequences of the impeded breathing. A very frequent complaint was the headache, which Meyer believed was caused by the pressure produced by the vegetations on the nerves of the roof of the pharynx. The affection of the ear was mainly caused by the position of the vegetation in the neighborhood of the Eustachian tube. The latter was the gravest among the symptoms, because of the liability to the development of serious disease of the middle ear. It seemed that chronic catarrh of the fauces was the most common cause of this affection, and climate the next. At least, the great number of cases which Meyer observed in Copenhagen spoke for its frequent occurrence in a cold and damp climate. It occurs most frequently between seven and fifteen years of age, and rarely in persons over twenty-five years old. Meyer advised operation as soon as possible, before the muscles of the palate have lost their normal tonus, so as to prevent permanent impairment of speech. In operating, he avoided those vegetations that are situated under the mouth of the Eustachian tube, fearing that he might lacerate the musculus salpingopharyngæus. Dr. Guleke had not seen harm attend the use of the galvano-caustic loop, although Meyer objected to its use, because of the danger involved in operating in a place which cannot be seen; but he had been compelled to proceed slowly, and with his finger in the immediate neighborhood of the loop.

In response to questions, Dr. Guleke remarked that he proposed to remove what remained of the tumor in the patient presented by scraping it away either with a sharp spoon in a bent handle, or with a spoon arranged so that it could be slipped over the finger. These growths are not attached to the bones, may spring from any part of the pharyngeal wall, and are entirely distinct from ordinary mucous polypi.

The President referred to a case in St. Luke's Hospital, in which Dr. George A. Peters removed a naso-pharyngeal tumor, which was so large that it projected from one of the nostrils and nearly filled the other. The first examination led to the conclusion that it was a small-celled sarcoma, but subsequently it was proved that it was a fibroma. He thought that the tumors in Dr. Guleke's cases were of unusual size for simple vegetations.

Dr. Lange thought that the tumor in Dr. Guleke's patient was about as large as a small hen's egg. To his finger it gave the sensation of a tumor which had been covered with degenerated mucous membrane. Perhaps the growth itself originated in some deeper structure.

Dr. Peters said that it felt very much like the tumor which he removed at St. Luke's Hospital.

Dr. Guleke said that the tumors in his other cases were larger than that in the patient presented. He thought they corresponded to the description given by Meyer, who had observed flat ones three centimeters in thickness, and pendulous ones three or four centimeters in thickness.

Dr. Lange suggested that tumors of that size might disappear spontaneously.

Dr. Guleke thought that they would, probably, and Meyer had stated that they doubtless would disappear after the age of twenty-five, but that they should not be allowed to remain, because of the liability to permanent impairment of hearing and speech.

Dr. McBurney remarked that he had seen adenoid growths in the upper and posterior part of the pharynx, but none of them had been larger than a small white bean. He had seen cases in which there were numerous growths, adenoid in character, but most of the tumors were very much smaller than those in Dr. Guleke's cases. He had treated several cases by pulling the small tumors off, and had seen them treated by the application of caustics.

Dr. E. L. Keyes referred to a case in which he supposed he had to deal with a simple mucous polypus that entirely filled one nostril, and caused bulging of the nasal bones, in a boy sixteen years of age. The mass presented at the nostril as a mucous polypus. After the patient was anæsthetized, he found that the growth was solid, and filled the entire right nostril posteriorly, and by conjoined manipulation with two fingers he succeeded in removing it. It consisted of two portions, one of which was about three-fourths of an inch in diameter, perfectly spherical, fleshy-looking, and from the end of this came a growth which exhibited the usual features of a mucous polypus. The more solid portion of the tumor was submitted to Dr. Heitzman for microscopical examination, who reported that it was an adenoma. The softer portion Dr. Keyes examined, and found it to be an unmistakable mucous polypus. The disease returned twice, and in each instance he removed the growth with the fingers. The patient at the last operation was about twenty-one years of age. He was of a strumous habit. His nose was permanently bulged on the affected side.

Dr. Guleke remarked that he had not seen any report of cases belonging to the class which he intended to describe, in which the tumor grew forward and occupied the nose.

Dr. Stimson recalled a microscopical examination which he made of the tumor first removed in Dr. Keyes' case, and recollected distinctly that he found nothing except fibrous tissue.

Dr. Briddon referred to a case in which he removed what he supposed was an ordinary mucous polypus. The patient had a fit of coughing, and threw out a tumor about three inches in length, with a very tender pedicle. To the end of which was attached a fibrous mass that had evidently grown from the superior meatus, turned around the vomer, and extended into the opposite nostril. It originated in the nasal fossa.

*Strangulated Inguinal Hernia. Reduced en Masse—Operation—Recovery.*—Dr. Erskine Mason narrated a case as follows: A man, aged fifty-five years, and single, gave a history of having first suffered from hernia in 1863, while in the army, and after lifting heavy weights. He had kept the hernia up part of the time with a pad and bandage, and lately had worn a truss, which was inefficient. On Saturday morning he was taken with diarrhœa, and after he had three or four movements his hernia came down and he was unable to reduce it. He suffered great pain in the region of the hernia, went to the station-house, and from there he was sent to Bellevue Hospital in an ambulance, where he arrived about three o'clock in the afternoon. The house surgeon said that he was not at that time suffering from the symptoms of a strangulated hernia. On examition, however, an incomplete hernia was found in the right iliac region about the size of an orange, which he tried to reduce by taxis, but failed. The patient was then placed in a hot bath, where he remained thirty minutes, after which a hypodermic injection of morphia was given, when the house surgeon again tried taxis, as also did other members of the staff, and manipulation was kept up for about fifteen minutes. The mass then began to recede at once from the inguinal canal, and at the same time the house surgeon noticed the development of a tumor of about the size of that to which taxis had just been applied, and dull upon precussion above and outside the internal ring. A bandage was applied, and a stimulating enema was given, which produced no movement of the bowels. On the following morning the man complained of considerable pain, and it was found that the hernia had again descended. Taxis was applied, and the tumor ascended as it had previously done, but vomiting supervened, pain returned and radiated over the abdomen. At five o'clock in the afternoon Dr. Mason saw the patient, whom he found much prostrated and suffering from severe pain in the abdomen, which was somewhat tympanitic. He found a swelling in the region above the internal ring, with slight fullness in the inguinal canal, and by forcing the finger into the canal, and at the same time pressing upon the tumor from above, he could just reach the coil of intestine.

The patient was put under the influence of ether, and Dr. Mason proceeded to operate. The sac contained a good deal of fluid, but it did not have a bad oder. At the neck of the sac a small knuckle of intestine was found, which was quite congested. He divided the neck of the sac and then proceeded to press against the intestine, when he found that it did not recede into the abdominal cavity. He drew the intestine down, but could not push back the part which protruded. The finger was then swept around the intestine, and in the posterior part of the neck of the sac a rent was found, which had permitted the coil of intestine to escape into the subperitoneal tissue, from whence it was gently drawn and readily passed into the abdominal cavity. The edges of the sac were united with catgut sutures. No unpleasant symptoms had followed the operation. The incision was made directly under the cord, and the inguinal canal was opened. A drainage-tube was placed to the outside, and about the neck of the sac. The method of closing the sac with catgut sutures was one adopted by Mr. Southam, who had reported in the last number of the *Lancet* six cases in which it had been practiced successfully.

[At the stated meeting, held March 22, 1881, Dr. Mason reported that the patient made a rapid and satisfactory recovery.]

The President referred to a case of hernia that had been reduced *en masse*, in which he succeeded, after making an incision through the upper part of the scrotum, and upward as high as the external abdominal ring, in seizing the sac, which was at the far end of the inguinal canal, with vulsellum forceps, and in causing the hernial tumor to redescend to the level of the scrotum. He then relieved the strangulation and reduced the hernia without injury to the aponeurosis of the oblique muscle. He was uncertain whether that could be done as a rule.

Dr. Mason referred to a case of femoral hernia, in which, after dividing the constriction, the sac and its contents slipped up, and then he pulled it down, opened the sac, and liberated the intestine.

Dr. Lange referred to a case of "reposition *en bloc*," made by the patient himself in the forcible attempt to reduce an incarcerated inguinal hernia. The inguinal canal was wide and completely empty. One could feel at the end of it a stretched band, representing, as shown during the operation, the spermatic cord, pulled upward and outward by the sac, which was laying in the retroperitoneal tissue a short distance above Poupart's ligament, and was slightly marked by an insignificant elevation of the abdominal wall above it. A longitudinal incision was made above Poupart's ligament, and it was found that the sac was pushed upward, making an acute angle at its attachment to the internal ring, the latter being displaced backward toward the peritoneal cavity, and allowing in this way the sac to slip in an upward direction behind the peritoneum. The sac was closed by suture and removed. The patient had a good and speedy recovery. Dr. Lange did not know whether or not the hernia had recurred. The patient has been advised to wear a truss for a larger hernia.

*Acute Septicæmia—Influence of Germs in the Putrefactive Process.*—Dr. L. A. Stimson narrated a case as follows: A man, aged twenty-eight years, was brought to Bellevue Hospital last July, having fallen from the roof of a four-story building while asleep. There was evidence of injury of the spine in the lumbar region; there were two small, round wounds at corresponding points on each side of the internatal fold near its upper extremity; slight extravasation of blood in the left foot, and rupture of the sheath of the tendon of the tibialis posticus, allowing that tendon to slip forward. There was loss of sensibility on the outer side of the left leg, and it extended partly over the dorsum of the foot. On the fourth day, without previous complaint referable to that region, Dr. Stimson found deep emphysematous crackling in the swollen foot, about its inner side. The patient had had a chill; his temperature was 104° F. Immediate amputation was advised and accepted, and performed through the condyles of the femur. The patient died on the second or third day following.

The case was interesting, *first*, because of the supposed influence of germs in the production of septicæmia. It was a case in which putrefactive changes took place without the presence of an external wound.

*Second*, because two somewhat similar cases—acute septicæmia—had been reported quite recently before the Clinical Society of London, in which immediate amputation was performed in one, and in the other twenty-four hours after the development of the first symptoms. In the first case the patient recovered; the second terminated fatally.

In the discussion which followed, the principle of immediate amputation was laid down—that is, that in all cases of spreading gangrene, amputation should be performed immediately without waiting even for consultation.

Dr. Lange remarked that the case reported by Dr. Stimson was interesting, because of the fact that putrefactive changes occurred in short a time without external wound. But the absence of an external wound does not exclude the presence of bacteria. It is quite common to find bacteria in large quantity in places far from the external surface, as, for instance, in acute osteo-myelitis. In order to be sure about the absence of germs, microscopical examination could not well be dispensed with.

Dr. Stimson thought that if bacteria could reach the inner parts of the body without external wound, the theory which claimed that the grave complications of surgical wounds were due solely to their entrance from without, was too exclusive.

Dr. Weir referred to two cases which he thought might throw some light upon the subject. One was a double compound fracture of the ankle-joint. He attempted to save the limb, and adopted Lister's method. The man did well for a time. Suddenly one leg became gangrenous, while the other was doing well, and the patient died. Prior to death the discharges were examined microscopically, and no evidence whatever of the existence of organisms was found.

The second case occurred in the New York Hospital. It was also a compound fracture of the leg in which gangrene occurred while under antiseptic treatment, but no bacteria could be found in the discharges.

Dr. Stimson thought that when the nutrition of the cellular elements of tissue were modified by a partial loss of vitality, or by changes in the vascular supply or innervation, the catalytic process carried on by them in the liquid in which they are bathed may be so modified as to result in the production of substances which are themselves poisonous.

A reason by analogy for this supposition is found in vinous fermentation. This is ordinarily due to the growth and multiplication of a germ ; but it is also known that under certain circumstances, such as the retention of a grape upon the vine after complete maturity, vinous fermentation may take place within the fruit by the continuing life of the cells of the fruit itself, without the aid of the specific germ. In like manner it is not unreasonable to suppose the cell of animal tissue, by continuing to live after the normal conditions of its existence have been modified, may be expected to have a correspondingly modified action.

Dr. Lange said in that case alcohol would be found in the grape.

Dr. Stimson said that it had been found, as shown by the experiments of Pasteur and two of his associates some eight or ten years ago.

*Blood from Beneath the Permanent Antiseptic Dressing.*—Dr. F. Lange presented a specimen of blood, with the following history: It was removed from a patient who two weeks ago submitted to amputation of the breast and removal of the contents of the axilla. The permanent antiseptic-dressing was applied. On account of the extremely nervous condition of the patient, he removed the dressing on the fifth day, when it was found that the wound had healed by first intention. In attempting to remove the dressing one of the flaps was raised, and hemorrhage occurred beneath it. The blood underwent the same changes as occur in cases of subcutaneous injury, but considerable remained, which was aspirated *six* or *seven* days afterwards. To ascertain its character, about two ounces of a dark, entirely fluid blood, were withdrawn, which was examined microscopically, and found to contain living bacteria. The number of the white blood-corpuscles was not increased. Some of the blood-globules, both red and white, were undergoing disintregation. A number of the red ones were almost colorless, hardly visible; in the majority they were stellated and shrivelled.

Dr. Lange thought that the bacteria entered during the operation and remained, but, because of the antiseptic measures, were of such low vitality that they were unable to act upon this mass of blood. The patient has remained entirely well.

*Idiopathic Pyopneumothorax.*—The president asked if any of the members had examined microscopically the fluid withdrawn from the chest in cases of idiopathic pyopneumothorax. A patient, with phthisical history, came under his care at Roosevelt Hospital, who entered for injury of the elbow, and on examination of the chest, the usual signs of pneumothorax, with effusion, were discovered. Amphoric breathing and succussion were well marked. Ten days ago he was aspirated, and seventy ounces of blood removed, which was free from putrefaction. Nine days afterward he was again tapped, and sixty ounces of yellowish, thin, purulent, odorless serum were withdrawn, which Dr. Sands examined very carefully with the microscope without detecting the presence of bacteria. Some of the pus-corpuscles were partly disintregated. He asked whether it was the rule in indiopathic pyopneumothorax for the fluid withdrawn from the chest to be free from odor? He had an impression that idiopathic pneumothorax, with empyema, was often marked by the absence of putrefaction in the effusion, the explanation being that the lung tissue through which the air has passed had filtered the air, and thereby prevented the entrance of germs.

The president further remarked that it was difficult to understand why, if the perforation of the lung no longer existed, and communication with the exterior had ceased, pneumothorax should continue at all; because in pneumothorax due to a penetrating wound of the pleura, the air frequently disappears in a very short time, provided the wound is carefully closed. It might be, however, that in cases of empyema the pleural surfaces were so altered as to have lost their absorbing power.—*Medical Record.*

